# Transcriptomic landscape of hepatic lymph nodes, peripheral blood lymphocytes and spleen of swamp buffaloes infected with the tropical liver fluke *Fasciola gigantica*

**DOI:** 10.1371/journal.pntd.0010286

**Published:** 2022-03-23

**Authors:** Rui-Si Hu, Fu-Kai Zhang, Qiao-Ni Ma, Muhammad Ehsan, Quan Zhao, Xing-Quan Zhu

**Affiliations:** 1 College of Life Science, Changchun Sci-Tech University, Shuangyang, Jilin Province, People’s Republic of China; 2 State Key Laboratory of Veterinary Etiological Biology, Key Laboratory of Veterinary Parasitology of Gansu Province, Lanzhou Veterinary Research Institute, Chinese Academy of Agricultural Sciences, Lanzhou, Gansu Province, People’s Republic of China; 3 Department of Parasitology, Faculty of Veterinary and Animal Sciences, The Islamia University of Bahawalpur, Bahawalpur, Punjab Province, Pakistan; 4 College of Veterinary Medicine, Shanxi Agricultural University, Taigu, Shanxi Province, People’s Republic of China; 5 Key Laboratory of Veterinary Public Health of Yunnan Province, College of Veterinary Medicine, Yunnan Agricultural University, Kunming, Yunnan Province, People’s Republic of China; Queen’s University Belfast, UNITED KINGDOM

## Abstract

The tropical liver fluke *Fasciola gigantica* is a parasitic helminth that has been frequently reported to infect mammals, typically involving water buffaloes. In this study, we characterized the tissue transcriptional landscape of buffaloes following infection by *F*. *gigantica*. RNAs were isolated from hepatic lymph nodes (hLNs), peripheral blood lymphocytes (pBLs), and spleen at 3-, 42- and 70-days post-infection (dpi), and all samples were subjected to RNA sequencing analyses. At 3 dpi, 2603, 460, and 162 differentially expressed transcripts (DETs) were detected in hLNs, pBLs, and spleen, respectively. At 42 dpi, 322, 937, and 196 DETs were detected in hLNs, pBLs, and spleen, respectively. At 70 dpi, 376, 334, and 165 DETs were detected in hLNs, pBLs, and spleen, respectively. Functional enrichment analysis identified upregulated immune-related pathways in the infected tissues involved in innate and adaptive immune responses, especially in hLNs at 42 and 70 dpi, and pBLs at 3 and 42 dpi. The upregulated transcripts in spleen were not enriched in any immune-related pathway. Co-expression network analysis further identified transcriptional changes associated with immune response to *F*. *gigantica* infection. Receiver operating characteristic (ROC) curve analysis showed that 107 genes in hLNs, 32 genes in pBLs, and 36 genes in spleen correlated with *F*. *gigantica* load. These findings provide new insight into molecular mechanisms and signaling pathways associated with *F*. *gigantica* infection in buffaloes.

## Author summary

*Fasciola gigantica* is a socioeconomically important tropical liver fluke of mammals, causing fascioliasis–a neglected tropical disease. In the present study, RNA sequencing and bioinformatic approach were employed to explore the global transcriptional changes of hepatic lymph nodes (hLNs), peripheral blood lymphocytes (pBLs), and spleen of water buffaloes during *F*. *gigantica* infection at 3-, 42-, and 70-days post-infection (dpi). The results revealed significant transcriptional upregulation of genes associated with innate and adaptive immune responses in infected hLNs (42 and 70 dpi) and pBLs (3 and 42 dpi). However, downregulation of transcripts involved in immune response was detected in pBLs at 70 dpi. The downregulated transcripts were enriched in metabolic pathways, such as drug metabolism-cytochrome P450 in infected hLNs at 3 dpi. These findings provide new insight into the pathogenesis of *F*. *gigantica* in its natural mammalian host.

## Introduction

*Fasciola hepatica* and *Fasciola gigantica* are members of the genus *Fasciola*. *F*. *hepatica* has a worldwide geographical distribution, while *F*. *gigantica* is found in tropical regions, including Asia, Africa, and some parts of Europe [1,2]. *Fasciola* spp. can infect between 2.4 and 17 million people, and 180 million are at risk of infection. *Fasciola* spp. also can infect a wide range of livestock animals, of which cattle and water buffalo are the most affected species [3,4]. While *F*. *hepatica* infects cattle species (*Bos taurus*) [5,6], buffaloes are often infected with *F*. *gigantica* in Asia and south China [7–10]. The pathology caused by *Fasciola* spp. infection mainly takes place in liver, and its severity depends on the parasite burden. The clinical symptoms of fascioliasis is divided into acute (e.g., anorexia, flatulence, hepatomegaly, and splenomegaly) and chronic (e.g., duct dilatation, cholangitis, liver abscesses, and cirrhosis) phases [11]. These symptoms can adversely affect the health and productivity of buffaloes, and even lead to death, resulting in significant economic losses.

Infection with *F*. *gigantica* occurs when animals ingest grass and water containing infective metacercariae (encysted juvenile parasites). After ingestion, the metacercariae excyst within the duodenum and develop into newly excysted juveniles (NEJs), which penetrate through the intestinal wall into the peritoneal cavity [4]. After 3~6 days post-infection (dpi), NEJs migrate through the liver capsule and parenchyma, and grow via feeding and burrowing through liver tissue. During this phase, the pathological features in infected liver are characterized by traumatic tissue damage (hemorrhage) and inflammatory response, and fibrosis at 5~6 weeks post-infection. After 7~8 weeks, the flukes enter the biliary ducts and establish a chronic phase, where they mature into adults at 8~10 weeks post-infection [8].

Despite significant efforts [7,9,12,13], knowledge about the immune responses in buffaloes experimentally infected with *F*. *gigantica* remains limited. Furthermore, *F*. *gigantica* infections are not only limited to the liver of the host but also involve other immune-related tissues. For example, hepatic lymph nodes (hLNs) adjacent to the liver are connected to lymphatic vessels and play important roles in protecting against liver diseases. The spleen, the largest lymphatic organ in the body, produces various immune substances such as immunoglobulins. The peripheral blood lymphocytes (pBLs), including B cells and T cells, counter infection by various pathogens. Previously, we have identified many differentially expressed proteins (DEPs) in *F*. *gigantica*-infected buffalo tissues, including hLNs, serum, and spleen, using iTRAQ proteomics [7,9]. However, the molecular mechanisms underlying the pathological effects of *F*. *gigantica* infection in these tissues at the transcriptional level remain largely unknown.

All helminth infections elicit Th2 regulatory immune response [14,15], which plays roles in toleration [16] and resistance [17] mechanisms during helminth infection. Tolerance mechanisms and resolution of pro-inflammatory response can reduce the helminth impact on host fitness without directly affecting the helminth burden. In contrast, resistance mechanisms can promote the expulsion of helminths and prevention of reinfection. Maintaining a balance between toleration and resistance is important for both host and parasite. However, such balance in *F*. *gigantica*-infected buffaloes is not fully known. In addition to Th2 response, mixed Th1/Th2 response, suppression of Th1/Th17 responses, and differential responses of immune cells (e.g., macrophage activation, eosinophil apoptosis, and dendritic cell maturation) have been reported in infected tissues in ruminants or rodents infected by *F*. *hepatica* [4,18,19]. However, the equivalent mechanisms in the context of *F*. *gigantica* infection are poorly understood.

In this study, transcriptomic responses of buffalo’s hLNs, pBLs, and spleen to experimental infection by *F*. *gigantica* were examined using RNA-sequencing (RNA-seq). Our comparative analysis revealed significant transcriptomic changes of buffalo tissues at 3, 42, and 70 dpi. We identified hundreds of differentially expressed transcripts (DETs), infection-specific patterns, and genes in different infected tissues that correlated with *F*. *gigantica* load.

## Materials and methods

### Ethics statement

Water buffaloes were handled in accordance with the Animal Ethics Procedures and Guidelines of the People’s Republic of China, and the study was approved by the Animal Ethics Committee of Lanzhou Veterinary Research Institute, Chinese Academy of Agricultural Sciences (Permit number: LVRIAE-2015-08).

### Experimental animals, *F*. *gigantica* infection and tissue sampling

Eighteen water buffaloes (8-10-months old) were purchased from a local water buffalo farm in Guangxi Zhuang Autonomous Region, China. Buffaloes were randomly assigned into 6 groups (3 buffaloes per group), including 3 infected groups and 3 uninfected groups (control group). Buffaloes were kept in a clean shed and provided with enough water and food. The fecal egg counting (FEC) sedimentation method was used to monitor the fluke burden in buffaloes, and serum samples were tested for anti-*Fasciola* IgG and IgM antibodies using an Enzyme-Linked Immunosorbent Assay (ELISA), as described previously [20]. Additionally, buffaloes received orally 1 mL of triclabendazole (5% per kilogram of body weight) to ensure the absence of existing or prior helminth infections.

After a 4-week of the drug withdrawal period, buffaloes in the three infected groups were inoculated orally with 500 viable metacercariae contained within digestible capsules in 1 mL 0.85% sodium chloride (NaCl) solution; buffaloes in the three control groups were mock-infected with an equal volume of 0.85% NaCl solution contained within digestible capsules but without metacercariae.

At 3, 42, and 70 dpi, the presence of *F*. *gigantica* in infected liver and bile duct was confirmed by examination, as described previously [8,9] and buffalo’s hLNs, pBLs and spleen tissues were collected. Each tissue was washed with PBS several times and put into sterile tubes and stored at -80°C, until use.

### RNA extraction and Illumina sequencing

The RNA of each sample was extracted using TRIzol Reagent (Invitrogen, CA, United States) following the manufacturer’s protocols. RNA samples were treated with 20 units of RQ1 RNase-Free DNase (Promega, Madison, USA) to remove residual genomic DNA. The purity (OD_260/280_) of the RNA preparation was examined using a NanoPhotometer spectrophotometer (IMPLEN, CA, United States), and all RNA preparations had an absorbance ratio OD_260/280_ > 1.8. The integrity and concentration of RNA were examined using RNA Nano 6000 Assay Kit (Agilent Technologies, CA, United States) and Qubit RNA Assay Kit (Life Technologies, CA, United States), respectively. Three micrograms of each RNA sample were used for the construction of RNA libraries according to the protocol of NEBNext Ultra RNA Library Prep Kit (Illumina, NEB, United States). Each library was sequenced on an Illumina HiSeq 2500 (Illumina, San Diego, CA, United States) resulting in standard pair-end 2 × 150 reads.

### Transcript prediction and homologous annotation

FastQC (v0.11.9; https://www.bioinformatics.babraham.ac.uk/projects/fastqc/) was used to process the raw data for quality control analysis. Low-quality reads and adapters were filtered using Trim_Galore (v0.6.2; https://github.com/FelixKrueger/TrimGalore) to generate clean reads. The reference genome, annotation information and transcript sequence of swamp buffalo were downloaded from Genome Warehouse of China (http://bigd.big.ac.cn/gwh/) [21] with accession number GWHAAJZ00000000. The genome of swamp buffalo is a high-quality reference genome that has been recently released [22]. The repetitive sequences of the genome and low-complex DNA sequences were masked using RepeatMasker v4.0.9_p2 [23], and on the basis of the masked buffalo genome (~ 47% repeat sequences), the comprehensive RNA-seq data were used to assemble RNA transcripts and alternative isoforms. We first used HISAT2 v2.2.1 [24] and StringTie v2.1.4 [25] to identify the initially annotated reference transcripts and perform novel transcript prediction. The transcript sequences were extracted from the buffalo genome using gffreads v0.12.1 [26], their protein-coding regions were predicted using TransDecoder v5.5.0 [27] and, simultaneously following the software strategy, the longest ORF sequences were screened out as candidate transcripts. To minimize false-positive transcripts caused by RNA-seq noise, we evaluated the transcripts based on their expression level, gene structure, protein-coding regions. Transcripts satisfying the following criteria were considered as a *bona fide* mRNA transcript: (i) at least one sample has transcript reads ≥ 30, and the average reads of a transcript in all samples ≥ 3; (ii) a transcript at least has 3 exons, and the sequence length is ≥ 500 bp; (iii) transcript has ≥ 50% sequence encoding a protein and the length of the protein sequence is ≥ 100 aa.

We used BRAKER2 tool to further improve the transcript predictions. BRAKER2 [28] is a new and fully automated transcript annotation pipeline that is able to use reference genome and RNA-seq data or related species protein data sets to perform gene prediction in eukaryotes, which is based on the combined training method by means of GeneMark and AUGUSTUS models. In this study, the masked genome and RNA-seq data (including hLNs, pBLs, and spleen) of buffalo, as well as the protein sequences of closely related *Bos taurus* were used as the input data of BRAKER2 program. The eventual results contained an AUGUSTUS GTF file. Transcript results from StringTie and AUGUSTUS were assembled into a GTF file using EVidenceModeler v1.1.1 [29], resulting in data with non-redundant gene structure. Finally, the known and homologous genes were identified by nucleotide alignment with the *Bos taurus* protein using BLASTp (E-value cutoff 1e-10).

### Differential expression and functional enrichment analyses

We quantified the expression of the identified non-redundant transcripts using StringTie v2.1.4 across all samples. The expressed read counts of each transcript were used for differential expression analysis using DESeq2 v1.30.1 package [30], and the screening threshold for DETs was *P*-value < 0.05 and |log2 fold-change| ≥ 1.5. Then, we performed Gene Ontology (GO) and Kyoto Encyclopedia of Genes and Genomes (KEGG) functional enrichment analyses following the strategy of TBtools software [31] to obtain the functional signatures of DETs. Briefly, there were three steps to perform GO enrichment analysis: (i) transcript sequences of buffalo were BLASTp with all Uniprot protein datasets (access on January 2021), and the best hits were considered as homologous protein; (ii) the corresponding GO terms for homologous protein names matching to buffalo’s transcripts were retrieved from the “idmapping.tar.gz” file available at ftp://ftp.pir.georgetown.edu/databases/idmapping/, and considered as the background list of GO enrichment analysis; (iii) based on the calculation principle of the hypergeometric distribution test, GO enrichment analysis of DETs was performed in TBtools program. For KEGG enrichment analysis, there were two steps: (i) online KAAS web-based server (https://www.genome.jp/tools/kaas/) was used to automatically annotate the KEGG orthologs (KO) of buffalo transcripts; (ii) KEGG enrichment analysis of DETs was performed using TBtools based on the principle of hypergeometric distribution test.

### Co-expression network and correlation analysis

We performed weighted gene co-expression network analysis (WGCNA) to identify the co-expressed gene modules and analyzed the relationship between gene network and phenotype to find the core genes in the network. Briefly, the WGCNA R package v1.0.1 [32] was used to establish a correlation matrix between buffalo transcript expression and *F*. *gigantica*-infected tissues. TPM (Transcripts per Million) expression matrix of transcripts was used as the input data. The soft-threshold power for 0.9 was chosen as the correlation coefficient threshold and the minimum number of genes in modules was set to 10. To merge similar modules, we defined 0.2 as the threshold value for cut height. Days post-infection and infection status (where the number 1 and 0 represent the infected group and control group, respectively) were used as input traits in the module-trait correlation analysis. The correlation clusters of interconnected genes that shared similar expression patterns were considered as co-expression modules, and high module groups with significance values greater than 0.5 were selected as predictors that may be correlative with *F*. *gigantica* load. In order to test the predictive performance between module hub genes and *F*. *gigantica* infection, pROC package v1.66.0 [33] was used to evaluate the performance of predictors based on receiver operating characteristic (ROC) curve, and to calculate the ROC curve (AUC) based on the expression comparison of a given gene identified in the significant module. A higher AUC means that a module gene expressed in the infected and uninfected tissues has a better classification. Finally, module genes related to *F*. *gigantica* infection in each tissue were selected out with gene AUC value > 0.6 and TPM > 1.

## Results

### Confirmation of *F*. *gigantica* infection in the infected buffalo tissues

At days 3, 42, and 70 after infection, *F*. *gigantica* was detected in the infected liver and/or bile duct tissues of the buffalo and the parasite samples from all three infection time points have been collected previously [8,34], which confirmed the infection status of buffaloes. No flukes were identified in the tissues of control animals and no eggs were detected in the control animals. In addition, ELISA serological results showed that IgG and IgM antibodies against *Fasciola* spp. were negative in control groups.

### Characteristics of the transcriptome dataset

The RNA integrity numbers (RINs) of each RNA template > 8.0. In this study, we generated 54 high-quality libraries, all of them were subjected to the paired-end Illumina sequencing, and each infected and control group contained 3 biological replicates. The read number (raw and clean reads), Q20, Q30, and GC content of quality control are shown in S1 Table and at least 37 million clean reads were generated in each library, of which > 99% of the reads showed high-quality value > Q20, > 88% of the reads were up to Q30, and the GC content was 49~55%. By genome-based prediction and functional annotation, we identified 21,659 transcripts; of which 16,460 transcripts shared high homology with the protein sequences of *Bos taurus* via BLASTp to the Uniprot data sets (E-value cut-off 1e-10).

**Fig 1:**
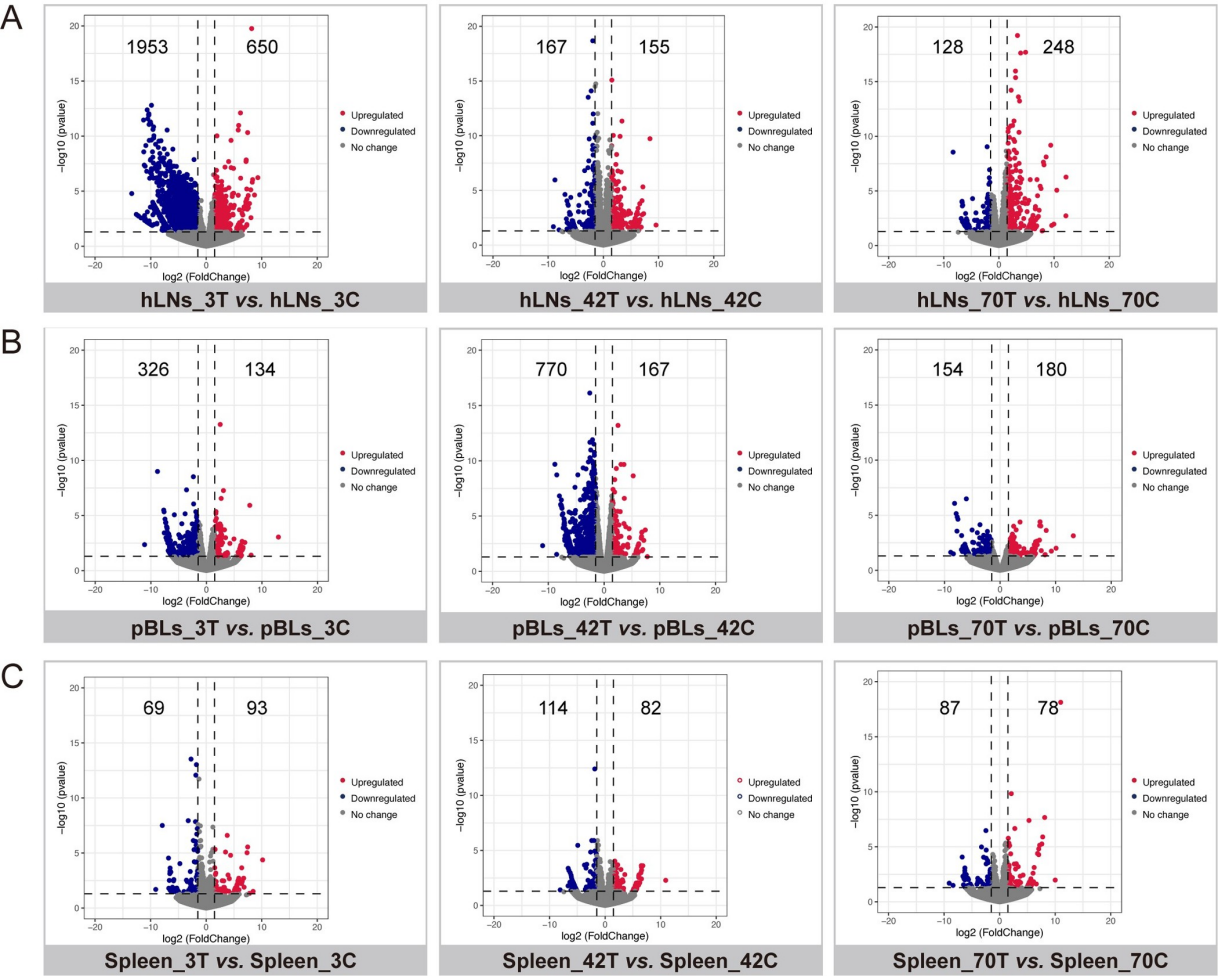
Volcano plots showing the differentially expressed transcripts (DETs) in different tissues of the buffaloes at 3-, 42- and 70-days post-infection (3, 42 and 70 dpi) (*P*-value < 0.05 and |log2 fold-change| ≥ 1.5). (A) DETs of hepatic lymph nodes (hLNs); (B) DETs of peripheral blood lymphocytes (pBLs); (C) DETs of spleen. Red dots represent the upregulated transcripts, blue dots represent the downregulated transcripts, and gray dots represent the unchanged transcripts in infected tissues.

Quantitative and differential expression analysis of transcripts (*P*-value < 0.05 and Log2FoldChange ≥ 1.5) revealed that: (i) in the infected hLNs, 650, 155, and 248 transcripts were upregulated at 3, 42, and 70 dpi, respectively, whereas 1953, 167, and 128 transcripts were downregulated at 3, 42, and 70 dpi, respectively (Fig 1A and S2 Table); (ii) in the infected pBLs, 134, 167, and 180 transcripts were upregulated at 3, 42, and 70 dpi, respectively, whereas 326, 770, and 154 transcripts were downregulated at 3, 42, and 70 dpi, respectively (Fig 1B and S2 Table); (iii) in the infected spleen, 93, 82, and 78 transcripts were upregulated at 3, 42, and 70 dpi, respectively, whereas 69, 114, and 87 transcripts were downregulated at 3, 42, and 70 dpi, respectively (Fig 1C and S2 Table). For DETs across all tissues, results showed that there were not any shared DETs across all tissues as mentioned in the black circle connected below the vertical line and the histogram (S1 Fig), indicating the lack of commonly shared DETs in hLNs, pBLs, and spleen, as the infection progresses.

### GO and KEGG enrichment analysis

We performed functional enrichment and pathway analyses of the DETs in infected tissues. The downregulation of transcripts in the infected hLNs at 3 dpi was significantly enriched for GO terms involved in the neuron or cell-related processes, such as neurogenesis, cell communication, neuron differentiation, and neuron projection development, while the upregulation of transcripts for GO terms was mainly involved in eukaryotic cell division, such as mitotic sister chromatid segregation, nuclear chromosome segregation, and mitotic nuclear division. Notably, in the upregulated transcripts in the infected hLNs at 42 and 70 dpi and the infected pBLs at 3 and 42 dpi, most of the enriched transcripts associated with GO terms or pathways were involved in the immune response. Typically, these included complement-mediated attack, mixed innate and adaptive immunity, and inflammatory response (S3 Table). It was noteworthy that the transcripts for immune-related GO terms or pathways, including response to stimulus, immune system process, and cytokine-cytokine receptor interaction were downregulated in the infected pBLs at 70 dpi. Additionally, we did not observe any immune-related processes or pathways for transcript changes that were significantly enriched in the spleen, and the downregulated transcripts of the spleen were also not significantly enriched in any GO term.

**Fig 2:**
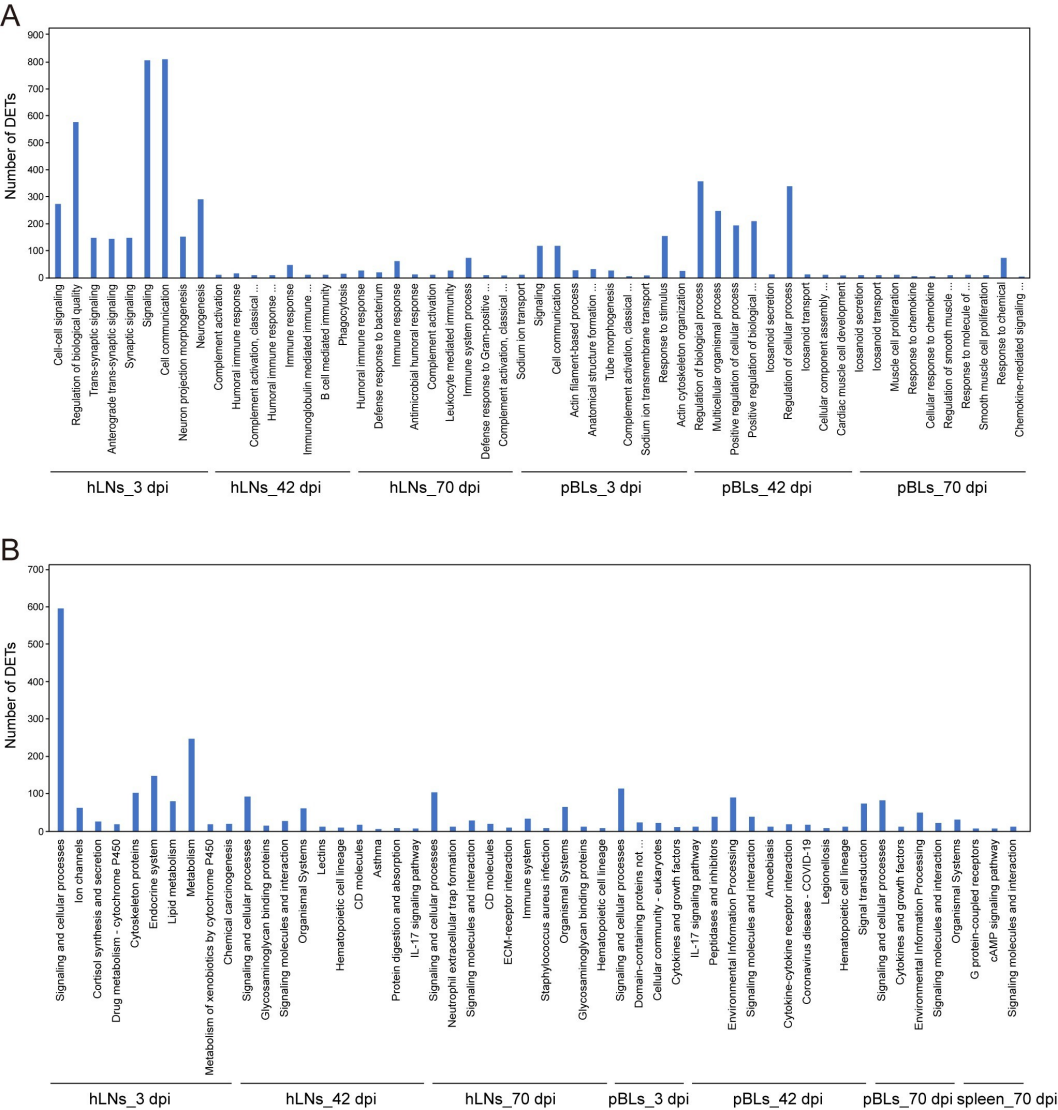
Bar plots showing the results of GO enrichment (A) and KEGG analysis (B) of DETs in tissues of the infected buffaloes.

**Fig 3:**
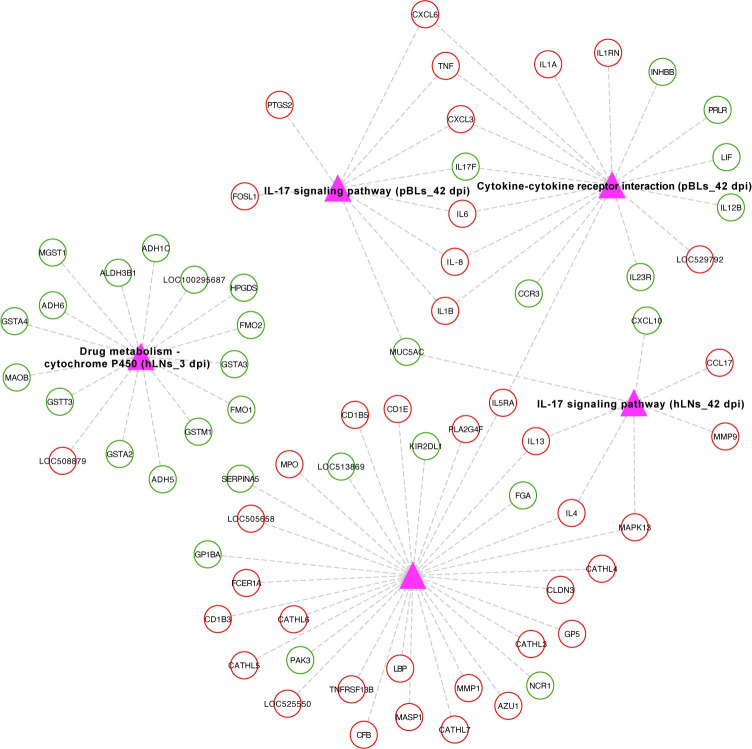
Differentially expressed components of four of interesting pathways involved in immune response and drug metabolism in *F*. *gigantica*-infected hLNs and pBLs. Pink triangle represents the pathway name, and circle represents the gene name, of which red circle represents the upregulated transcripts in infected tissues and blue circle represents the downregulated transcripts.

We also performed GO and pathway analysis of all DETs. According to the corrected *P*-value < 0.05, the top 10 significantly enriched GO terms are shown in Fig 2A. These terms included the involvement of both hLNs and pBLs at 3, 42, and 70 dpi, while the dysregulated transcripts of the spleen were not significantly enriched in any GO term. Similarly, the top 10 significantly enriched pathways are shown in Fig 2B. These included DETs associated with hLNs and pBLs at 3, 42, and 70 dpi, and spleen at 70 dpi. In terms of these pathways, we selected four of interest including drug metabolism-cytochrome P450 (hLNs_3 dpi), IL-17 signaling pathway (hLNs_42 dpi and pBLs_42 dpi), immune system (hLNs_70 dpi), and cytokine-cytokine receptor interaction (pBLs_42 dpi) to show the related genes and their expression changes in the infected tissues (Fig 3). From the results, it was noteworthy that 15 out of 16 genes involved in drug metabolism-cytochrome P450 and genes partially associated with immune pathways can be downregulated, suggesting the importance of biological changes caused by the parasite in different infected tissues.

### Co-expression network and module-trait functional annotation

**Fig 4:**
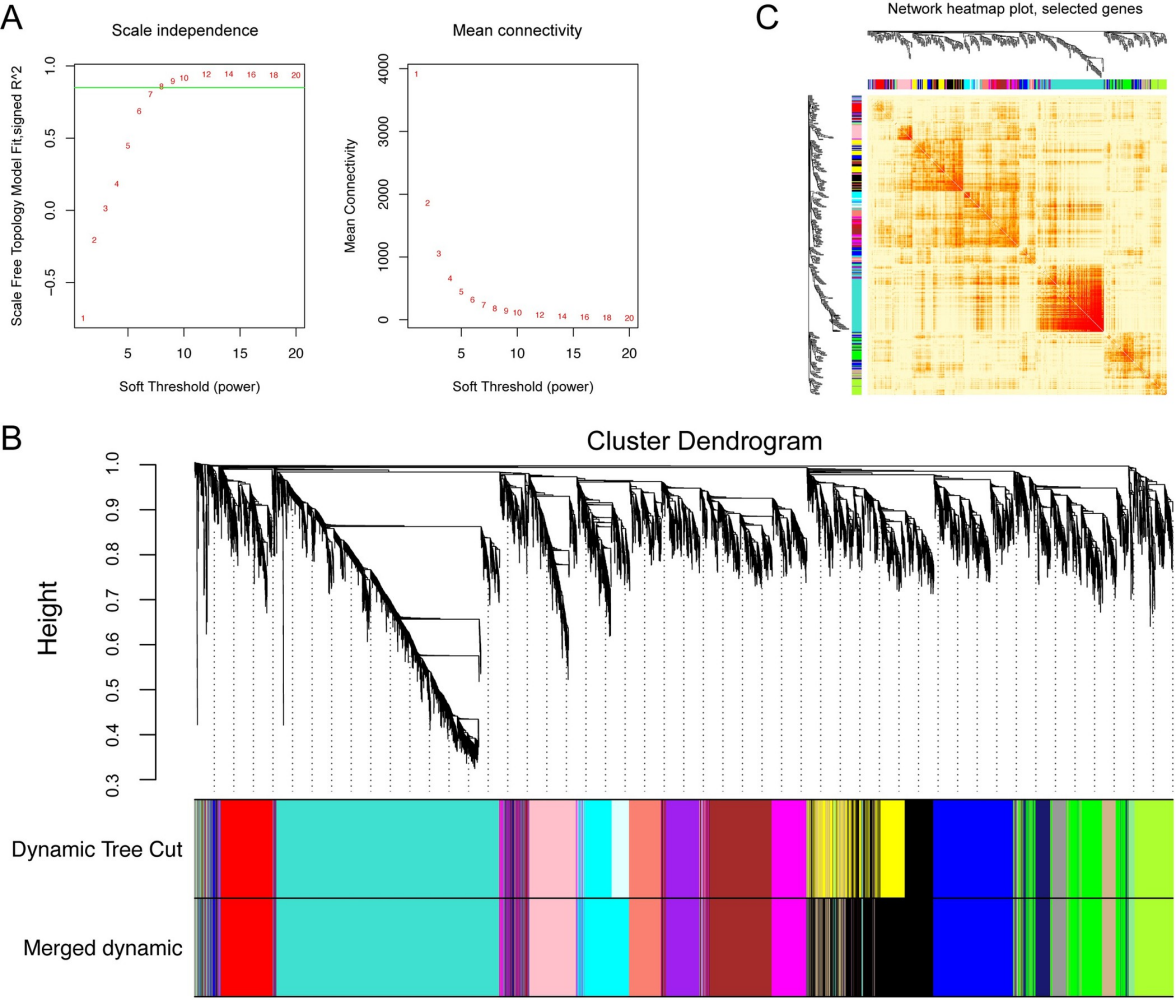
WGCNA analysis revealed the gene co-expression network of hLNs, pBLs and spleen at 3, 42 and 70 dpi, respectively. (A) Analysis of the scale-free network analyzed the weight of soft thresholds (left) and the average connectivity of various soft thresholds (right). (B) Cluster dendrogram of hLNs, pBLs and spleen genes. (C) Network heatmap of 400 randomly selected genes in co-expression module (the red gradient indicates the higher overlap area of functional module).

**Fig 5:**
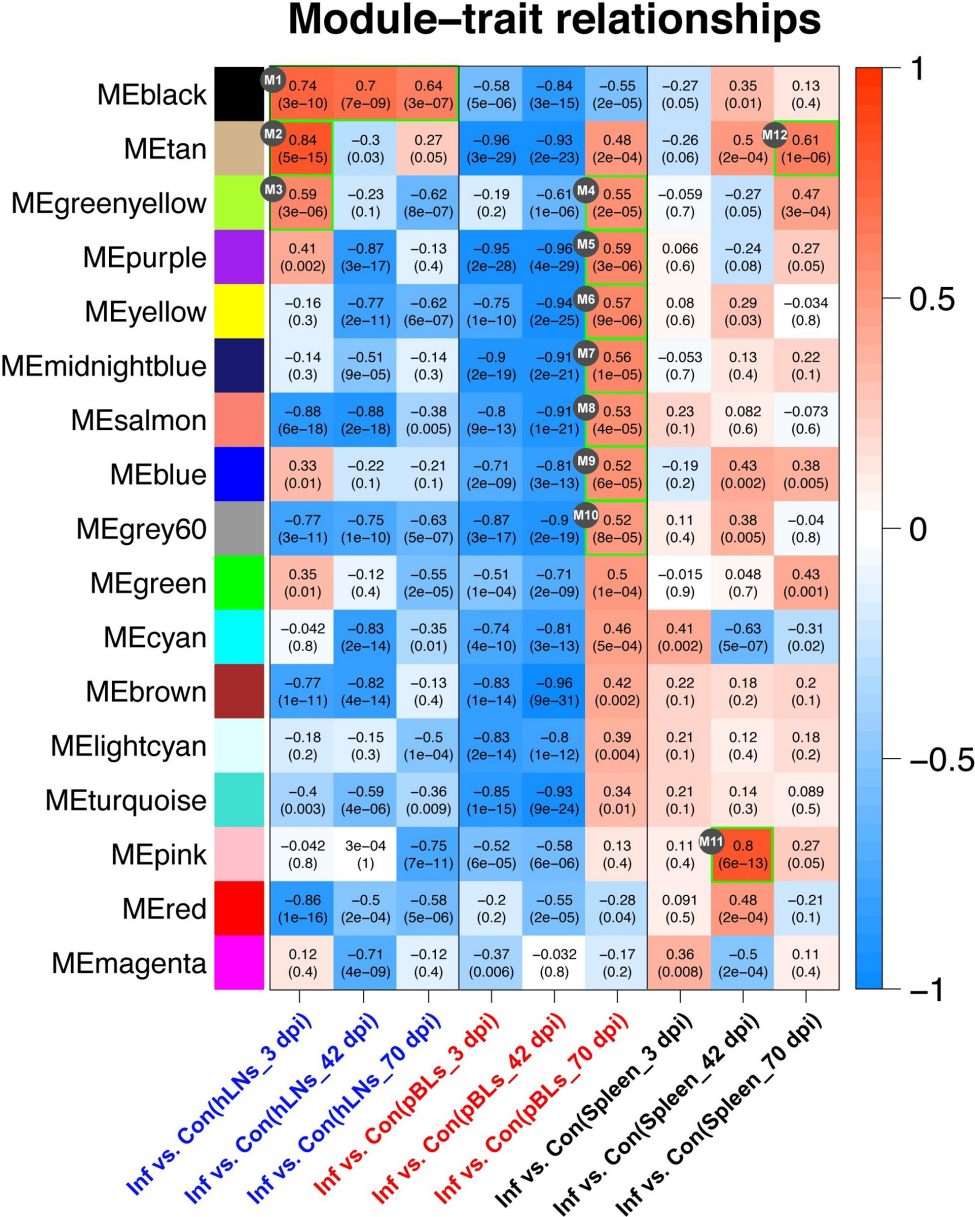
Module-trait relationship analysis of comparison groups (infected *vs*. control) in hLNs, pBLs and spleen. The significant modules with value > 0.05 are marked by green box, namely M1 to M12.

When a scale-free topology fit index of 0.9 was used as the correlation coefficient threshold, the soft-threshold power was selected as seven to construct WGCNA (Fig 4A). As shown in Fig 4B, 17 co-expression modules were constructed. The module comprising most genes was the turquoise module, followed by the blue module, brown module, and red module. Randomly selected genes of 400 were used for the topological overlap heatmap analysis, and the results showed that those modules comprising most genes were independent of other modules (Fig 4C). Module-trait correlation analysis showed that multiple modules were related to *F*. *gigantica* infection and, with regarding the hLNs, pBLs, and spleen tissue at the three infection time points, the module-trait correlation with value > 0.5 was considered as further analysis. Among these, 3 modules (M1~M3) were from the hLNs group, 7 modules (M4~M10) from the pBLs group, and 2 modules (M11 and M12) from the spleen group (Fig 5). All genes from these modules were selected out and could be considered as hub genes.

To explore the functional characteristics of the potential hub genes, we performed GO enrichment analysis for these 12 module genes and found that genes from the M1, M2, M3, M4, M11, and M12 were significantly enriched in immune-related biological processes. In the hLNs, the M1 module was highly correlated to all three infection time points (i.e., 3, 42, and 70 dpi), and genes were significantly enriched in the immune system process, immune response, and defense response (S2A Fig). However, the M2 and M3 module genes were only significantly related to 3 dpi, of which M2 module genes were significantly enriched in leukocyte activation, lymphocyte activation, and immune system development (S2B Fig), while M3 module genes were significantly enriched in the regulation of response to stimulus, regulation of immune response, and leukocyte mediated immunity (S2C Fig). In the pBLs, the M4 module was significant at 42 dpi, their genes were significantly enriched in the regulation of response to stimulus, regulation of immune response, and leukocyte-mediated immunity (S2D Fig). In the spleen, the M11 module was significant at 42 dpi, their genes were involved in T cell activation and differentiation (S2E Fig). While the M12 module was significant at 70 dpi, their genes were significantly enriched in lymphocyte activation, lymphocyte-mediated immunity, and adaptive immune (S2F Fig).

### Validation and efficacy evaluation of hub genes

**Fig 6:**
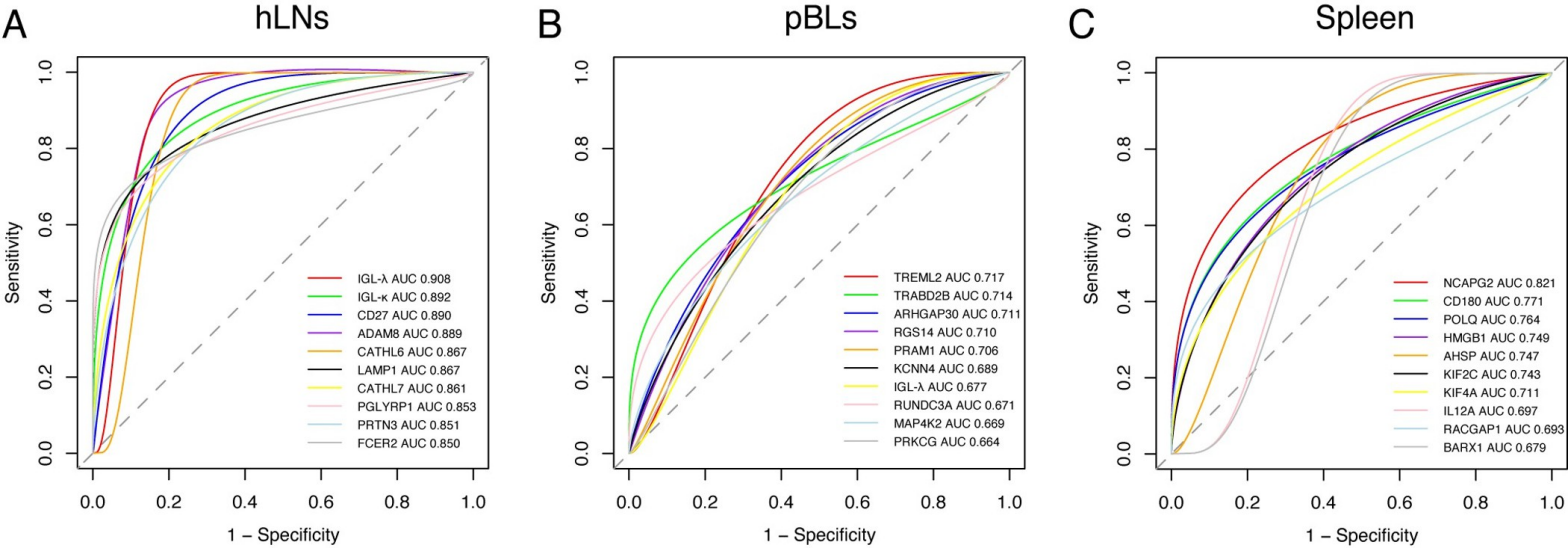
ROC curve analysis revealed hub genes associated with *F*. *gigantica* infection. The plot showing the top 10 hub genes with AUC value > 0.6 of hLNs (A), pBLs (B) and spleen (C).

Results of the ROC analysis revealed the validation of hub genes from the WGCNA modules, of which there were 107 genes in the hLNs, 32 genes in the pBLs, and 36 genes in the spleen that were predicted to be correlated with *F*. *gigantica* load. The details of these genes including areas under the ROC curve (AUC), chromosome position, differential expression analysis for each time point, and function description are summarized in S4 Table. As shown in Fig 6, ROC analysis revealed the top 10 hub genes of significant modules in the hLNs (Fig 6A), pBLs (Fig 6B), and spleen (Fig 6C). By comparing the top 10 genes in these three tissues, the AUC values of all genes from hLNs were ≥ 0.85, which were obviously higher than that of other genes in the pBLs and spleen (majority of them < 0.8), indicating a high correlation between hLNs and *F*. *gigantica* infection.

## Discussion

In this study, we performed a comparison of transcriptomic changes in three important immune tissues (hLNs, pBLs, and spleen) of buffaloes following the infection by the liver fluke *F*. *gigantica* using RNA-seq approach. Differential expression analysis of transcripts between infected and uninfected tissues revealed significant changes in the number of DETs at 3, 42, and 70 dpi (Fig 1). *F*. *gigantica* infection altered the expression of hLNs transcripts with more than 2,000 DETs at 3 dpi, compared with the other two infection time points and other two tissues. These transcriptional changes in hLNs at 3 dpi exhibited a similar profile to the differentially expressed proteins (DEPs) identified in *F*. *gigantica*-infected buffaloes [9]. These results suggest that early infection and migration of *F*. *gigantica* NEJs from the intestinal wall into the peritoneal cavity are associated with significant transcriptional dysregulation in hLNs.

We have previously presented a detailed proteomic analysis of buffalo’s hLNs, global serum, and spleen following the infection of *F*. *gigantica* at 3, 42, and 70 dpi [7,9]. By comparing the association between transcripts and proteins in the hLNs, global serum (containing the component of pBLs) and spleen, we identified 558 DETs that could be translated into 536 unique proteins, and 209 unique proteins were DEPs (S2 Table). The differences between DETs and DEPs may occur during the progression from gene transcription to protein translation. Additionally, among the corresponding DETs and DEPs, we noted that most of them had the same expression pattern, simultaneous upregulation or downregulation expression signature (S2 Table), indicating a biological relevance between transcripts and corresponding proteins.

At 3 dpi in hLNs, upregulation of immune-related biological processes or pathways in infected animals was not significant. Interestingly, we observed significant downregulation of metabolism-related processes, such as metabolism, lipid metabolism, and drug metabolism-cytochrome P450 (S3 Table). The drug metabolism-cytochrome P450 pathway has been previously shown to be significantly downregulated in *F*. *gigantica-*infected liver tissue [9]. Here, we found 15 genes involved in the drug metabolism-cytochrome P450 pathway that were downregulated in infected animals, including 7 glutathione S-transferases (*GSTA2*, *GSTA3*, *GSTA4*, *GSTM1*, *GSTO2*, *GSTT3*, and *MGST1*), 4 alcohol dehydrogenases (*ALDH3B1*, *ADH1C*, *ADH5*, and *ADH6*), 1 flavin-containing monooxygenase (*FMO1*), 1 hematopoietic prostaglandin (*HPGDS*), 1 glutathione transferase (*LOC100295687*), and 1 amine oxidase (*MAOB*) (Fig 3). Interestingly, *GSTM1*, *GSTT3*, *ADH5*, and *MAOB* were also significantly downregulated at the protein level in infected animals (S2 Table). Among these genes, glutathione S-transferases (GSTs) play roles in regulating detoxification enzymes [35]. In mammalian tumor cells, elevated expression of GSTs is implicated in resistance to chemical carcinogens and anti-cancer agents [36]. Hence, it could be assumed that downregulation of various GSTs in infected animals may reduce the resistance to early infection and lead to adverse reactions or therapeutic failures.

In the hLNs, at 42 and 70 dpi, immune-related processes or pathways such as immunoglobulin response, humoral immune, adaptive immunity, B cell-mediated immunity and cytokine-receptor interaction were significantly upregulated in infected animals (S3 Table). There were three immunoglobulin-related genes (*IGL-λ*, *IGL-κ*, and *IGHM*) and two Th2-type cytokines (*IL-4* and *IL-13*) significantly upregulated (> 4-fold) in infected animals. The WGCNA analysis showed that the transcriptional changes of these genes in infected animals are associated with *F*. *gigantica* load (S4 Table). Previous studies have shown that overexpression of immunoglobulins plays a role in countering the invasion of *Fasciola* spp. [37–40]. Th2-type immune response results in humoral immune responses and produces helminth-specific antibodies [41]. The expression of *IL-4* and *IL-13* activates B-cell proliferation, Ig class-switching, IgE antibody production, and impels IgE-Fc receptors cross-linking and binding to the helminth surface [42,43]. Fc receptors that bind to IgE during interaction with the helminth surface are divided into two types: high-affinity Fc epsilon R1 and low-affinity Fc epsilon R2 [44]. In our data, expression of a high-affinity Fc receptor (*FCER1A*) at 3 and 70 dpi was significantly upregulated in infected animals, but without any corresponding change in the proteome results. KEGG pathway enrichment analysis suggested that Fc epsilon RI signaling pathway was significantly upregulated (S3 Table). Interestingly, expression of another high-affinity Fc receptor (*FCER1G*) was inhibited in chronic *F*. *hepatica* infection (16 weeks) in sheep [45], but the expression of *FCER1G* was not altered in the present study. Because polymorphism of Fc receptor exists in many organisms [46], the role and relevance of Fc receptor-mediated IgE regulation in *Fasciola*-infected hosts should be elucidated in further studies.

In infected animals, pBLs had significant upregulation of Th2/Th17 type immune response at 3 and 42 dpi; however, Th1-type immune was suppressed at 70 dpi (S3 Table). At 3 dpi, pBLs exhibited a high expression level of humoral and adaptive immune responses, such as complement activation and leukocyte migration to counter the parasite invasion (S3 Table), and most immune-related genes were upregulated in infected animals. However, we observed that Th17-related genes (*IL-17F*, *IL-17RD*, *IL-23*, and *IL23R*) were significantly downregulated, which is similar to previous findings on *F*. *hepatica*-induced infection in ovine peripheral blood mononuclear cells [18]. Moreover, the other two genes, *GHSR* (growth hormone secretagogue receptor) and *SFRP1* (secreted frizzled-related protein 1), that are involved in the growth and regulation of immune cells such as macrophages, T cells, and B cells, were also downregulated significantly by 9 and 100-fold, respectively. *GHSR* can promote the switch of macrophage phenotype to anti-inflammatory M2 in mice [47]. The M2 macrophages promote the resolution of inflammation and secretion of anti-inflammatory mediators [48]. Therefore, downregulation of *GHSR* during early infection stage may not be helpful to the resolution of pro-inflammatory, thereby increasing the helminth impact on host fitness. *SFRP1* can respond to *IL-1* during the immune response, and negatively regulates the proliferation of B cells. The downregulation of *SFRP1* in infected animals indicates that blood cells during early stages of infection preferentially activate humoral immunity to resist the parasite invasion. Interestingly, the new coronavirus disease (COVID-19) related pathway was found activated (Fig 2B). Helminth infection can induce a controlled inflammatory component to increase host Th2-type response, thereby alleviating the invasion of a novel coronavirus [49]. Therefore, the activation of this pathway during *F*. *gigantica* infection further supports the involvement of Th2-immune response during *F*. *gigantica* infection.

The upregulated transcripts of pBLs at 42 dpi were involved in inflammatory response, and some genes were significantly upregulated, including four cytokines (*IL-1A*, *IL-1B*, *IL-6*, and *IL-8*), and two neutrophil-related chemokines (*CXCL3* and *CXCL6*), and one nitric oxide synthase (*NOS2*). *NOS2* encodes inducible nitric oxide synthase (iNOS) and produces a carbon monoxide (NO) molecule, which has microbicidal and antiparasitic properties [50]. During the inflammatory response, NO can increase the synthesis and expression of inflammatory cytokines, such as *IL-1*, *IL-6*, and *IL-8* [51,52]. This result is consistent with a previous observation of increased expression of *NOS2* and inflammatory mediators (*IL-1A*, *IL-1B*, *IL-6*, *IL-8*, *CXCL3*, and *CXCL6*) in PBMCs of *F*. *hepatica*-infected cattle [53]. However, regulation and alternative activity of iNOS can be suppressed, such as in *F*. *hepatica*-infected ovine PBMCs [18] and *F*. *hepatica* antigen (fatty acid-binding protein)-induced human macrophages [54]. Therefore, this result suggests that regulation of *NOS2* may be species-specific and/or tissue-specific during *Fasciola* spp. infection.

The cellular immune response of pBLs at a late stage of infection (70 dpi) exhibited a reverse trend, and anti-pathogen immunity and immune system were downregulated. Previous studies have shown that infections induced by *Fasciola* spp. suppresses Th1-type cellular immunity [55,56]. The most representative Th1 type cytokines include *IL-12B* and *TNF* and their expressions were downregulated by 6 and 8-fold in infected animals, respectively. *TNF* induces dendritic cells to produce *IL-12*, which further induces Th1-type innate and adaptive immune responses. Antigen-presenting cells (APC) secrete *IL-12* and stimulate natural killer cells (NK) to produce *IFN-γ*, and then activate *STAT1* and promote the differentiation of Th1-type cells thereby mediating innate immunity, however adaptive immunity requires *IL-12* to activate *STAT4* [57]. Among the molecules that are involved in Th1-type response, the average expression values (TPM) of *IFN-γ*, *STAT1*, and *STAT4* in infected tissues were 0.85, 134, and 27, respectively. The expression value of *IFN-γ* was relatively low, which may play a role in the suppression of Th1-type immunity.

Although the spleen is the largest immune organ in the body, upregulation of transcripts during the infection did not fully activate the immune response pathways and the results of our study are very similar to our previous spleen proteomics study [9]. At 3 dpi, the number of spleen’s DETs was small, and not significantly enriched in any biological processes and pathway. At 42 dpi, we noticed that downregulated transcripts of infected spleen were enriched in biological processes related to immune regulation, such as suppressing innate immune response of the spleen. We identified 6 molecules involved in innate immune response, including Fc-γ receptor gene (*IFCGR1A*), 2’-5’ oligoadenylate synthase gene (*OAS1Y*), RNA helicase gene (*DDX58*), interferon-induced GTP-binding protein 2 gene (*MX2*), mannan-binding lectin serine peptidase 1 gene (*MASP1*), and interferon alpha-inducible protein 6 gene (*IFI6*). These genes were all downregulated in infected spleen at least by 4-fold. Since the spleen is not the direct target organ of *F*. *gigantica* infection, it is speculated that the reason may be related to products of *F*. *gigantica* that circulate in the body to the spleen tissue, thereby inhibiting the expression of these immune-related genes. Further research is needed to explore how the fluke effectors influence gene regulation in spleen, and the associated mechanisms of adaptive immune response. At 70 dpi, upregulation and downregulation of spleen transcripts were not enriched in any biological processes, indicating that the effect of *F*. *gigantica* on the spleen may be minimal at this phase of infection.

## Conclusions

In this study, RNA sequencing was used to characterize the transcriptional changes of three important immune-related tissues (i.e., hLNs, pBLs, and spleen) of water buffaloes during infection by *F*. *gigantica* at 3, 42 and 70 dpi. Many differentially expressed transcripts (DETs) were identified in infected tissues, with the largest change occurred in hLNs, especially at 3 dpi (> 2,000 DETs). Infection induced significant upregulation of genes and pathways, involved in innate and adaptive immune responses in infected tissues, especially in hLNs at 42 and 70 dpi, and pBLs at 3 and 42 dpi. Consistent with our previous proteomics study, spleen tissue did not exhibit a strong immune response against infection. WGCNA analysis identified many hub genes from co-expression modules that cause differential expression. Validation of the hub genes revealed that 107 hLNs genes, 32 pBLs genes, and 36 spleen genes were correlated with *F*. *gigantica* load. These findings improve our understanding of the immunopathology of *F*. *gigantica* infection in the buffalo host.

## Supporting information

S1 Fig The UpSet plot shows aggregation and intersection of DETs in hLNs, pBLs, and spleen at 3, 42 and 70 dpi. The yellow bar indicates the number of DETs in tissues of the infected buffaloes, the green bar indicates the number of intersecting DETs, the black circle at the bottom indicates the DETs presented in infected tissue, and the connecting lines indicate the intersecting organs or tissues.
(TIF)

S2 Fig GO functional enrichment analysis demonstrates that six gene modules, i.e., M1 (A), M2 (B), M3 (C), M4 (D), M11 (E) and M12 (F), are significantly enriched in immune-related processes.
(TIF)

S1 Table Quality characteristics of transcriptome paired-end sequencing results. (XLSX)

S2 Table The summary of differentially expressed transcripts (DETs) in tissues of the buffaloes at 3, 42 and 70 dpi, as compared with previous proteomic investigations. (XLSX)

S3 Table The summary of GO enrichment and KEGG analysis of differentially expressed transcripts in tissues of the infected buffaloes. (XLSX)

S4 Table ROC curve analysis of module genes (AUC value > 0.6). (XLSX)
