# Supplementary figures and images for: Transcriptomic landscape of hepatic lymph nodes, peripheral blood lymphocytes and spleen of swamp buffaloes infected with the tropical liver fluke Fasciola gigantica
Source: PLoS Negl Trop Dis. 2022 Mar 23;16(3):e0010286. doi: 10.1371/journal.pntd.0010286 (PMC8942208; doi:10.1371/journal.pntd.0010286)

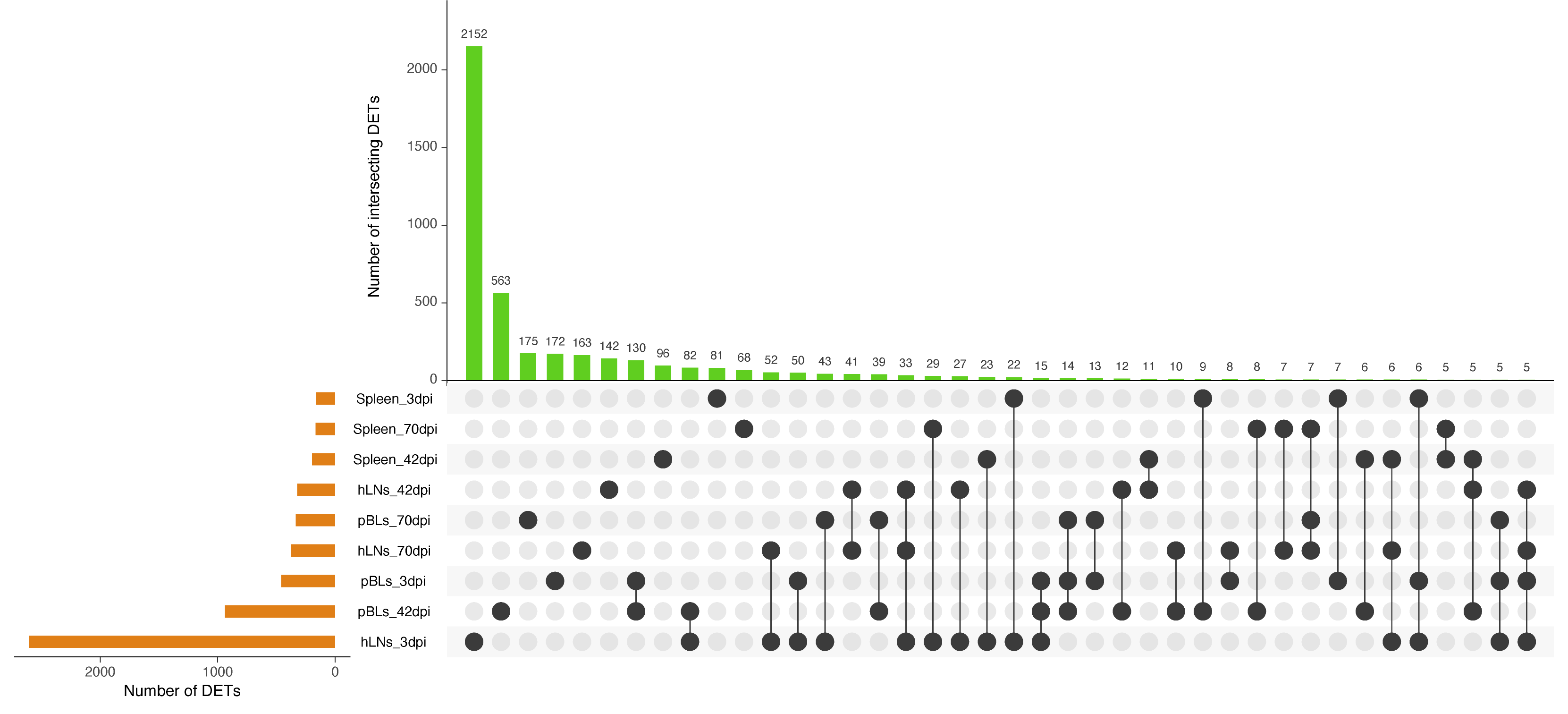

Supplement: S1 Fig — The yellow bar indicates the number of DETs in tissues of the infected buffaloes, the green bar indicates the number of intersecting DETs, the black circle at the bottom indicates the DETs presented in infected tissue, and the connecting lines indicate the intersecting organs or tissues. (TIF) [file pntd.0010286.s001.tif]

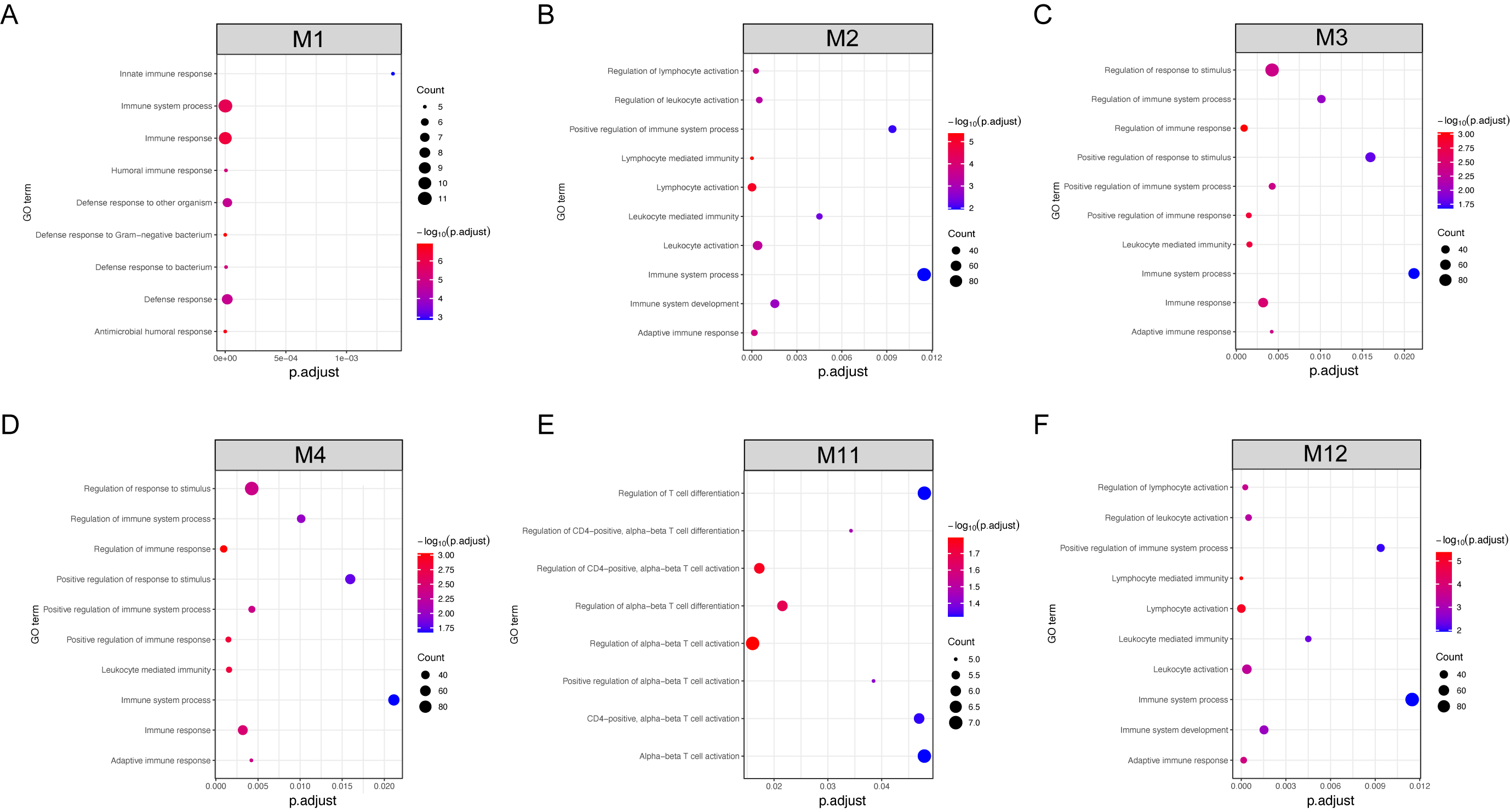

Supplement: S2 Fig — GO functional enrichment analysis demonstrates that six gene modules, i.e., M1 (A), M2 (B), M3 (C), M4 (D), M11 (E) and M12 (F), are significantly enriched in immune-related processes. (TIF) [file pntd.0010286.s002.tif]
